# Mapping the intellectual structure and landscape of nano-drug delivery systems in colorectal cancer

**DOI:** 10.3389/fphar.2023.1258937

**Published:** 2023-09-14

**Authors:** Yonglong Chang, Qinling Ou, Xuhui Zhou, Kechao Nie, Haixia Yan, Jinhui Liu, Jing Li, Sifang Zhang

**Affiliations:** ^1^ Department of Integrated Traditional Chinese and Western Medicine, The Second Xiangya Hospital, Central South University, Changsha, China; ^2^ National Clinical Research Center for Metabolic Diseases, Changsha, China; ^3^ Department of Addiction Medicine, Hunan Institute of Mental Health, Brain Hospital of Hunan Province (The Second People’s Hospital of Hunan Province), Changsha, China; ^4^ College of Integrated Traditional Chinese and Western Medicine, Hunan University of Traditional Chinese Medicine, Changsha, China

**Keywords:** colorectal cancer, nano-drug delivery system, bibliometric analysis, visualization, R-bibliometrix, research trends

## Abstract

**Background:** Colorectal cancer (CRC) is a prevalent malignancy affecting the digestive tract, and its incidence has been steadily rising over the years. Surgery remains the primary treatment modality for advanced colorectal cancer, complemented by chemotherapy. The development of drug resistance to chemotherapy is a significant contributor to treatment failure in colorectal cancer. Nanodrug delivery systems (NDDS) can significantly improve the delivery and efficacy of antitumor drugs in multiple ways. However, there is a lack of visualization of NDDS research structures and research hotspots in the field of colorectal cancer, and the elaboration of potential research areas remains to be discovered.

**Objective:** To comprehensively explore the current research status and development trend of NDDS in CRC research.

**Methods:** Bibliometric analysis of articles and reviews on NDDS for CRC published between 2002 and 2022 using tools including CiteSpace, VOSviewer, R-bibliometrix, and Microsoft Excel was performed.

**Results:** A total of 1866 publications authored by 9,870 individuals affiliated with 6,126 institutions across 293 countries/regions were included in the analysis. These publications appeared in 456 journals. Abnous Khalil has the highest number of publications in this field. The most published journals are the International Journal of Nanomedicine, International Journal of Pharmaceutics, and Biomaterials. Notably, the Journal of Controlled Release has the highest citation count and the third-highest H-index. Thematic analysis identified “inflammatory bowel disease”,“ “oral drug delivery," and “ulcerative colitis” as areas requiring further development. Keyword analysis revealed that “ulcerative colitis,” “exosomes,” and “as1411”have emerged as keywords within the last 2 years. These emerging keywords may become the focal points of future research.

**Conclusion:** Our findings reveal the current research landscape and intellectual structure of NDDS in CRC research which helps researchers understand the research trends and hot spots in this field.

## 1 Introduction

Colorectal cancer (CRC) imposes a substantial burden as one of the leading causes of cancer-related mortality worldwide (Choukaife et al., 2022; Siegel et al., 2023). It primarily affects the colon and rectum as a malignancy, being linked to the inactivation of the p53 pathway or adenomatous polyposis coli gene, as well as the accumulation of mutations in genes such as K-ras, dysregulation of the transforming growth factor-beta pathway, and the formation of small polyps (Radaic et al., 2020). The Globocan report, issued by the International Agency for Research on Cancer (IARC), provides data on the incidence, mortality, and survival rates of different cancer types in different regions and countries. This information can aid healthcare professionals, researchers, policymakers, and the public in gaining an understanding of the global burden of cancer distribution. Additionally, it contributes to the development of appropriate policies for cancer prevention, screening, and treatment. The latest Globocan report predicts that by 2035, the number of new colorectal cancer cases is expected to reach 2.5 million worldwide, surpassing the prevalence of other more common cancers, such as stomach and liver cancers. (Yu et al., 2015; Chang et al., 2023). Nevertheless, despite continuous advancements in the diagnosis and treatment of primary and metastatic colorectal cancer, long-term survival and cure rates persist disappointingly low.

The American Joint Committee on Cancer has categorized CRC into five stages, each requiring specific treatment strategies (Yang and Merlin, 2020). Stage 0 is defined by the presence of abnormal colonic cells or mucosal polyps, and early surgical resection is highly effective in achieving a cure. Additionally, surgical resection is the recommended treatment for most cases of stage I-II, resulting in postoperative 5-year survival rates ranging from 37% to 74%. However, advanced CRC is associated with a significantly reduced survival rate, as low as 6%, necessitating the need for adjuvant medical treatment, such as postoperative chemotherapy. In stage III-IV, adjuvant chemotherapy is frequently employed in clinical treatment, utilizing agents such as oxaliplatin, 5-fluorouracil, cisplatin, doxorubicin, and other emerging drugs. Regrettably, the efficacy of these drugs is not consistently reliable, and their administration is often accompanied by adverse effects, including vomiting, hair loss, and nausea. Moreover, anticancer drugs have long been hampered by inherent limitations, such as inadequate hydrophobicity, low water solubility, insufficient biodistribution, and susceptibility to multidrug resistance (Yang and Xie, 2021). Consequently, there is an urgent need for innovative strategies that enhance the physicochemical and pharmacodynamic characteristics of conventional chemotherapeutic agents and enable targeted drug delivery. These approaches aim to optimize therapeutic effectiveness while simultaneously minimizing off-target side effects (Arumov et al., 2021).

**FIGURE 1:**
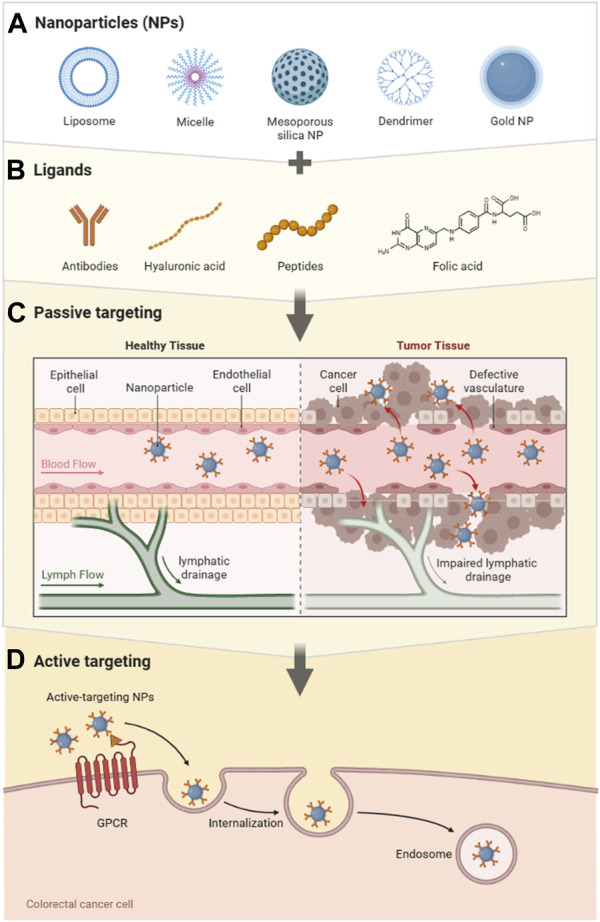
Schematic representation of nano-drug delivery systems in colorectal cancer. **(A)** Nanoparticles. **(B)** Ligands. **(C)** Passive targeting. **(D)** Active targeting.

Nanomaterials possess unique characteristics, including a small volume, extensive surface area, and high permeability (Lammers et al., 2012), that facilitate their effective integration with diverse biological materials (Pei et al., 2022). This integration enhances biocompatibility while enabling precise control over drug release (C. de S. L. Oliveira et al., 2021). Consequently, nanotechnology holds great promise as a valuable tool in cancer treatment. Substantial progress has been achieved in the past few decades regarding the clinical adoption of nanotechnology through the development of nanoparticle-based drug delivery systems (NDDS) (Kaushik et al., 2022). These systems can protect encapsulated drugs from degradation during circulation, facilitate targeted drug delivery, mitigate systemic toxicity, enhance drug solubility, and optimize both drug pharmacokinetics and therapeutic efficacy. NDDS has shown notable efficacy in domains where molecular targeted therapy encounters substantial limitations. This circumstance presents a compelling opportunity to expand the treatment options for CRC. The mechanism of action of NDDS in CRC is visually depicted in Figure 1 (Zhao et al., 2022).

Currently, bibliometrics has become a vital methodology for scholars to effectively identify the latest progress in research fields, predict hotspots, and evaluate trends (Zhu et al., 2019). In the context of nanomaterials, bibliometrics has been extensively applied. However, there is a lack of bibliometric analysis concerning the application of nanomaterials in oncology, where the interplay between oncology, materials science, pharmacology, and other disciplines is increasing (Maulvi et al., 2021). To the best of our knowledge, no bibliometric analysis specifically focuses on NDDS for CRC. Thus, this study employs the Web of Science Core Collection (WOSCC) database, along with CiteSpace, VOSviewer, and R-Bibliometrix, to conduct comprehensive bibliometric and visual analyses of NDDS for CRC literature (van Eck and Waltman, 2009). This analysis includes examining publication and citation counts, research trends in countries/regions, institutions, authors, and keywords. The aim is to identify research hotspots, predict development trends, and fill the existing knowledge gap in this field.

## 2 Methods

### 2.1 Data collection and search strategy

We performed a thorough search in the WOS Core Collection database (WOSCC) to identify publications about NDDS for CRC from 2002 to 2022. To conduct the search, we implemented wildcards in a systematic and scientific approach. Specifically, we used the following search strategy: (TS=((Nanoparticle* OR Nanocrystalline Material* OR Nanocrystal* OR Nano Particle*) AND (Drug Delivery System* OR Drug Targeting* OR Drug Delivery)) AND TS=(“Rectal Neoplasm” OR “Rectal Tumor” OR “Rectal Cancer” OR “Rectum Neoplasm” OR “Rectum Cancer” OR “Cancer of the Rectum” OR “Cancer of Rectum” OR “Colorectal Neoplasm” OR “Colorectal Tumor” OR “Colorectal Cancer” OR “Colorectal Carcinoma” OR “Colonic Neoplasm” OR “Colon Neoplasm” OR “Cancer of Colon” OR "Colon Cancer” OR “Cancer of the Colon” OR “Colonic Cancer” OR “CRC”)). We retrieved data on 16 April 2023, to ensure consistent results and avoid any changes due to daily updates.

**FIGURE 2:**
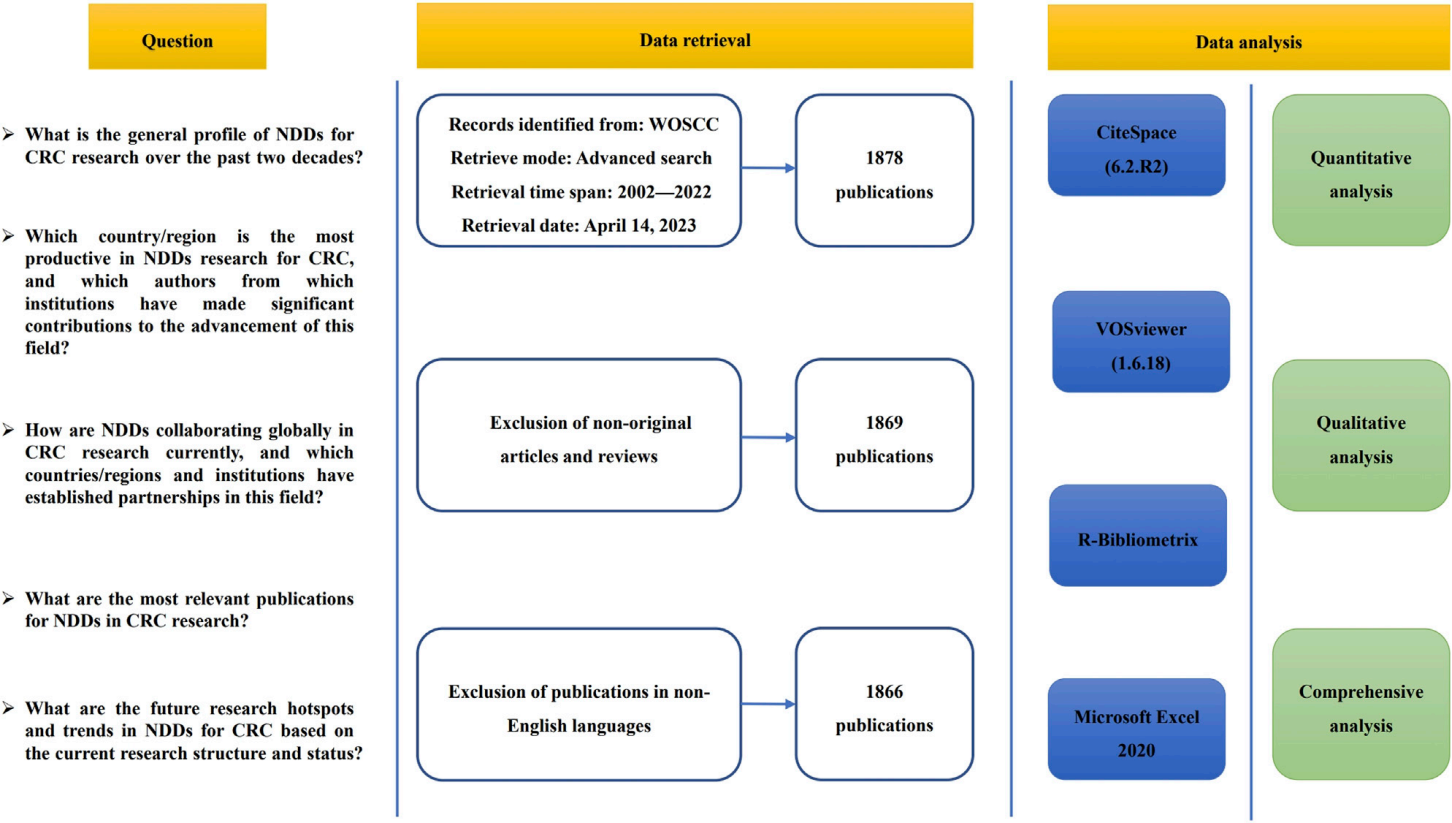
Flow chart of literature screening.

For our analysis, we considered only original articles and reviews published in English-language journals. We evaluated each publication’s relevance based on its title and abstract, and reviewed the full text of any unclear publications. Figure 2 presents the flowchart of our study.

### 2.2 Data analysis

#### 2.2.1 Quantitative analysis

To quantify the data, we will focus on four aspects: annual publication volume and its trend, top 20 countries/regions ranked by publication volume, top 10 journals ranked by publication volume, and top 10 authors ranked by publication volume.

#### 2.2.2 Qualitative analysis

Our analysis will address the following aspects: the average number of citations per year, journals and co-cited journals, authors and co-cited authors, co-cited references analysis, and co-cited keywords analysis.

#### 2.2.3 Comprehensive analysis

Visual analysis of collaboration between different countries/regions, h-indicators of top 20 journals, h-indicators of top 20 authors, research trends over the past 2 decades, and cluster analysis.

### 2.3 Visualization analysis

Bibliometric analysis was first conceptualized in 1969 by Alan Pritchard, a British intelligence scientist (Poly et al., 2023). Currently, bibliometrics has become an essential tool for monitoring the development and trends of publications (Jung et al., 2018). Bibliometric visualization software enables publication data extraction and analysis and facilitates the creation of knowledge graphs. This software can identify influential authors, key research topics, and collaboration patterns within a given field, providing insights for researchers, policymakers, and stakeholders.

In this study, we utilized several software tools for bibliometric analysis and visualization. Specifically, we employed CiteSpace (version 6.1) (Pan et al., 2018), which was developed by Prof. Chen C for bibliometric analysis and visualization. We also utilized VOSviewer (version 1.6.18) (van Eck and Waltman, 2009), a widely used software for building collaborative, co-citation, and co-occurrence networks. The visualization map of VOSviewer represents each node as a labeled circle, where larger circles indicate higher frequency in co-occurrence analysis, and the color of each circle is determined by the cluster they belong to. The thickness and length of the links between nodes reflect the strength and relevance of the connections between corresponding nodes (Peng et al., 2022). Up to 1,000 links can be set to show the strongest links between nodes. Additionally, we used R (version 4.1) package “bibliometrix” (https://www.bibliometrix.org) for data analysis visualization, including analyzing relationships between authors, institutions, and national collaborations, examining current research structures and future research trends, and identifying changes in research hotspots. Also, we utilized Microsoft Excel 2010 to create quantitative visualizations of certain data. By using these software tools, we were able to extract key information from numerous publications and generate visual maps that offered valuable insights into our research.

## 3 Results

### 3.1 Trend of annual publications and citations of NDDs in CRC

**FIGURE 3:**
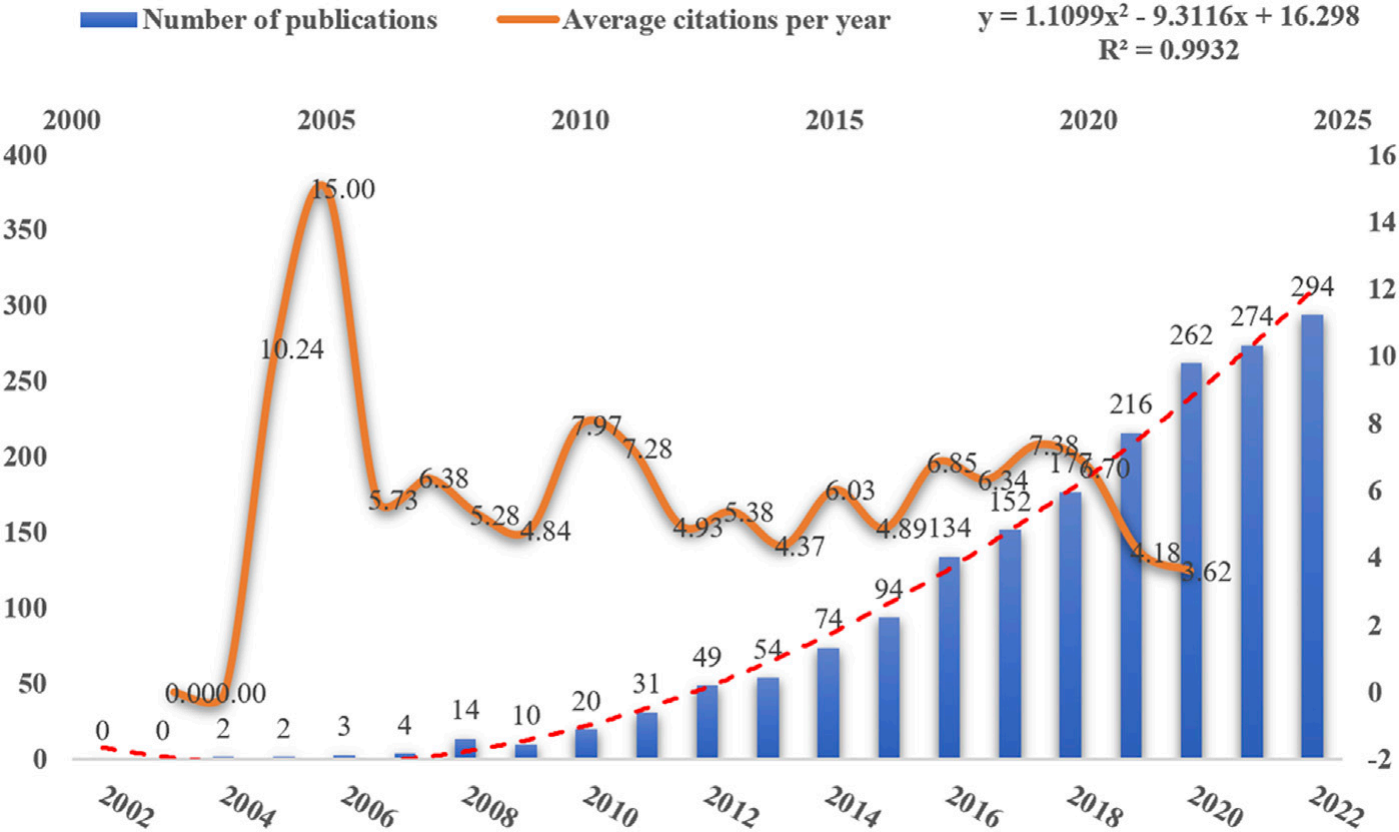
Trends in the volume of publications and average citations per year of NDDs in CRC.

The number of publications and their citation rates are useful indicators for monitoring research trends. In this study, as shown in **Figure 3**, we retrieved 1,866 publications related to NDDS research in CRC from the WoSCC database, consisting of 1,641 papers (87.94%) and 225 review papers (12.06%). The number of publications on this topic started with only 2 papers in 2004 and showed a significant upward trend, increasing from 20 in 2010 to 294 in 2022. Regarding average annual citations, the papers received an average of 10.24 citations in the year after the first 2 publications in 2004, with a peak of 15 citations in 2005. However, the average annual citations fluctuated after 2005 and were on average 28.01 citations per paper. Overall, the findings indicate that research on NDDS in CRC has been growing steadily over time, with a marked increase since 2010. This suggests that there is a growing interest among researchers in exploring the use of NDDS in CRC.

### 3.2 Country/region and institutional publication volume and cooperation network analysis

**FIGURE 4:**
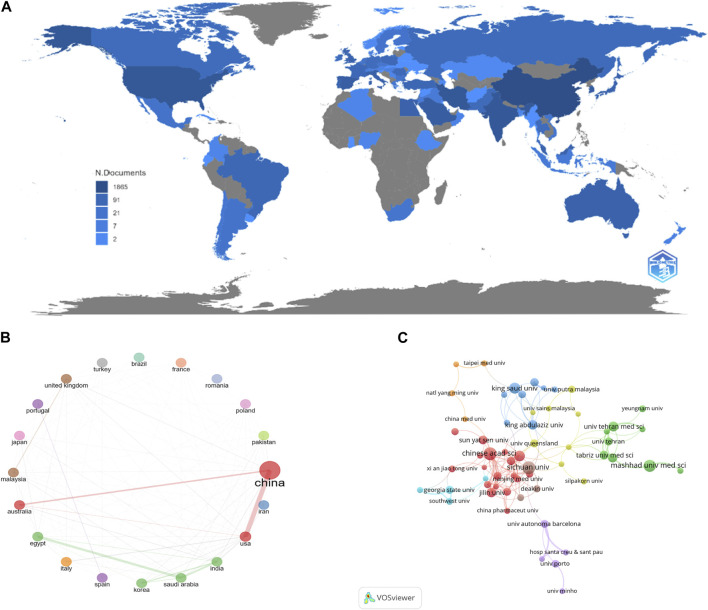
**(A)** Country/region number of publications. **(B)** Network of cooperation among top 20 countries/regions by number of publications. **(C)** Network of cooperation among top 20 countries/regions by number of publications.

A total of 6,126 institutions from 293 countries/regions have contributed to the research on NDDS in CRC. The number of publications from each country/region is presented in Figure 4A, with China ranking first with 605 publications, accounting for 32.42% of the total, followed by India with 268 publications, the United States with 267 publications, Iran with 173 publications, and Saudi Arabia with 111 publications. Table 1 shows the top 20 countries/regions in terms of the number of publications. Notably, despite ranking third in the number of publications, the United States has the highest intermediary centrality at 0.41, indicating the superior quality of their research in this field. Among the top 20 countries/regions, as shown in Figure 4B, China has established close cooperation with the United States and Australia, while Saudi Arabia, Egypt, and India have established partnerships with each other. However, some countries in the top 20 still conduct independent research in this field.

**TABLE 1:** Top 20 active countries/regions.

Rank	Country	Records	Centrality	Rank	Country	Records	Centrality
1	China	605	0.03	11	England	62	0.12
2	India	268	0.14	12	Malaysia	58	0.12
3	USA	267	0.41	13	Japan	57	0.07
4	Iran	173	0.24	14	France	39	0.13
5	Saudi Arabia	111	0.12	15	Portugal	37	0.07
6	South Korea	109	0.09	16	Turkey	35	0.02
7	Egypt	86	0.11	17	Brazil	30	0.07
8	Australia	78	0.05	18	Germany	30	0.13
9	Spain	70	0.17	19	Pakistan	28	0.09
10	Italy	66	0.09	20	Poland	26	0.12

The collaborative network depicting the contributions among institutions is displayed in Figure 4C. After importing the data into VOSviewer and setting the minimum number of publications for institutions to 10, a total of 69 institutions meet the criteria. These 69 institutions are then divided into 8 clusters. According to Table2, out of 6,126 institutions from 293 countries/regions that contributed to NDDS research in CRC, the Chinese Academy of Sciences had the highest number of publications (n = 42), followed by Sichuan University (n = 39), Mashhad University of Medical Sciences (n = 36), King Saud University (n = 32), and Zhejiang University (n = 26). Notably, three out of the top five institutions with the highest number of publications are from China, and 10 out of the top 20 institutions are also from China, indicating the significant efforts and contributions of Chinese research institutes in this field. Mediation between centrality is a crucial indicator for evaluating the significance of nodes in collaborative networks. Nodes with a mediated centrality value of 0.1 are considered important. Among the top 20 institutions with the highest number of published papers, three institutions have mediated centrality values greater than 0.1, including the Chinese Academy of Sciences (0.12), King Abdulaziz University (0.12), and Shandong University (0.14).

**TABLE 2:** Top 20 active institutions.

Rank	Institution	Records	Centrality	Rank	Institution	Records	Centrality
1	Chinese Academy of Sciences	42	0.12	11	Fudan University	19	0.05
2	Sichuan University	39	0.04	12	Tabriz University of Medical Sciences	19	0.05
3	Mashhad University of Medical Sciences	36	0.01	13	Shanghai Jiao Tong University	18	0.05
4	King Saud University	32	0.1	14	Georgia State University	18	0.01
5	Zhejiang University	26	0.03	15	Cairo University	17	0.1
6	Tehran University of Medical Sciences	22	0.05	16	Sun Yat Sen University	16	0.01
7	Jilin University	21	0.02	17	Xi An Jiao Tong University	15	0.01
8	King Abdulaziz University	21	0.12	18	Natl Taiwan University	15	0.03
9	Islamic Azad University	21	0.02	19	University of Granada	15	0
10	Imam Abdulrahman Bin Faisal University	21	0.03	20	Shandong University	14	0.14

### 3.3 Authors and co-cited authors

**FIGURE 5:**
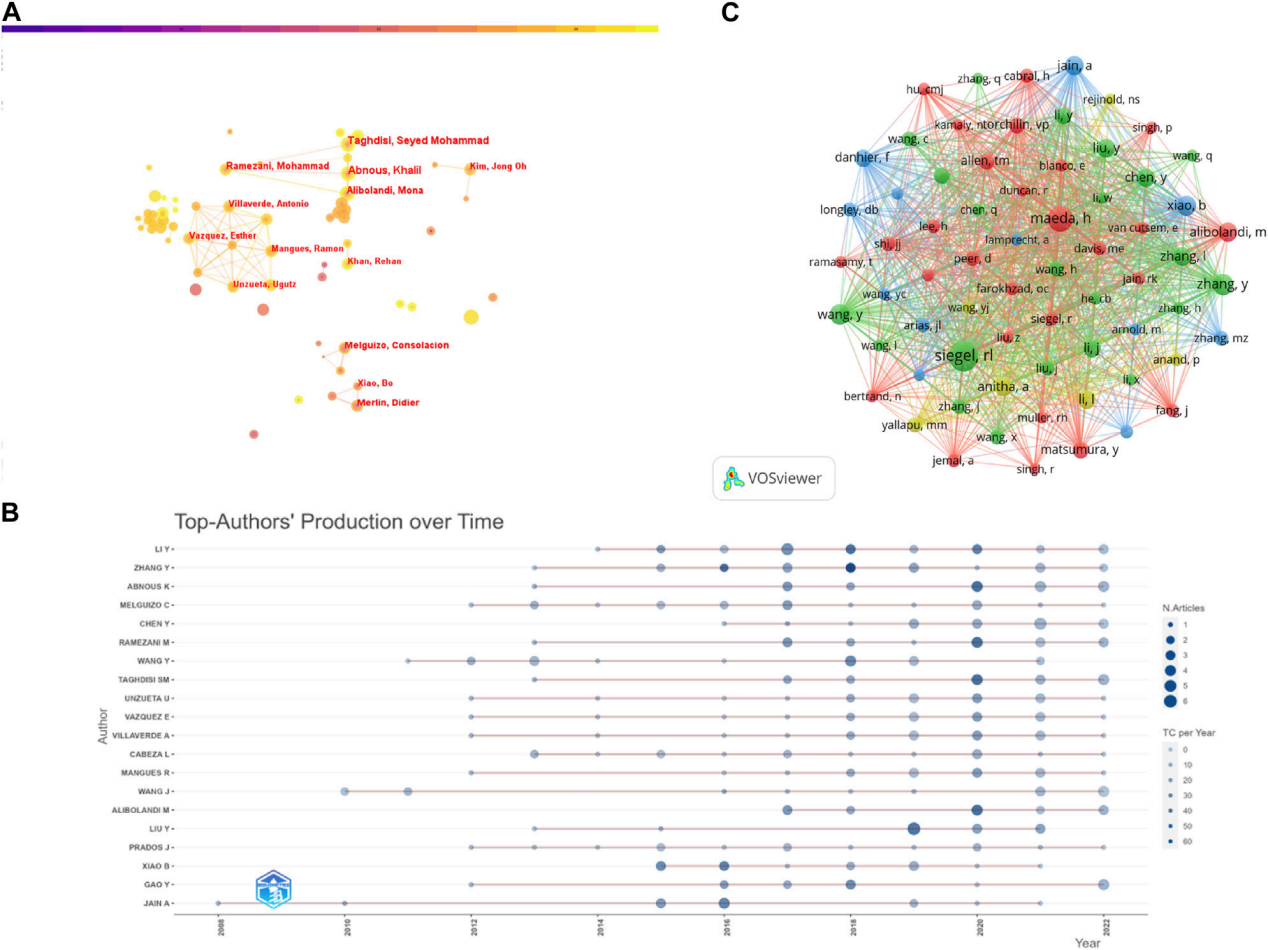
**(A)** Visual Analysis of Author Collaboration Using CiteSpace. **(B)** The relationship between the number of publications by the top 20 authors and temporal changes. **(C)** Visual Analysis of Author Co-citation Network Using VOSviewer.

An analysis of 1,866 publications revealed a total of 9,870 researchers contributing to the study of NDDS in CRC. Six of the top 10 authors had 10 or more publications each. To construct the collaborative network of authors, we used CiteSpace on the 1,866 peer-reviewed publications considered for analysis, resulting in 648 nodes and 1,044 links. The size of the nodes reflects the number of publications by each researcher, and more links between nodes indicate increased collaboration between authors in this field. As illustrated in Figure 5A, Abnous Khalil, Taghdisi Seyed Mohammad, Alibolandi Mona, Ramezani Mohammad, and Merlin Didier have the largest nodes due to their high number of relevant publications. Furthermore, we observed close cooperation between several authors.

We utilized the "bibliometrix" package of R (version 4.1) to generate a graphical representation of the relationship between the number of publications and time for the top 20 authors, as depicted in Figure 5B. The circle’s size corresponds to the number of publications per year, and its color represents the total number of citations per year. A darker shade of blue indicates a higher total number of citations per year. Most authors show a gradual increase in the number of publications over time. In 2020, 12 out of the top 20 authors produced more than 3 publications, with the highest total number of citations being 41. It is noteworthy that all four researchers, Abnous Khalil, Ramezani Mohammad, Taghdisi Seyed Mohammad, and Alibolandi Mona, had 41 total citations in 2020. Additionally, Liu Y set a new record for the highest number of publications in a single year during 2008–2022 with 6 publications in 2019.

Out of the 51,221 co-cited authors, eight were cited more than 100 times, as shown in Table 3, with the highest being Professor Siegel RL (n = 262), who is the director of the American Cancer Society’s Surveillance Information, Surveillance and Health Services Research Division. Her team has conducted extensive research on disparities in cancer incidence and mortality, with a specific focus on CRC, particularly early-onset disease. Her research contributed to the change in the age at which the American Cancer Society recommends starting colorectal cancer screening from 50 to 45 years of age, and she also serves as a scientific advisor for research programs in this area. Mmeda H and Zhang Y ranked second and third with 182 and 129 co-citations, respectively. To map the co-citation network, the authors with at least 50 co-citations were filtered, resulting in 67 eligible co-cited authors clustered into four groups centered on Siegel RL, Mmeda H, Zhang Y, and Wang Y, based on the Association strength algorithm. The co-cited authors under different clusters actively collaborated with one another. The co-citation network is illustrated in Figure 5C.

**TABLE 3:** Top 10 authors in terms of the number of publications and top 10 authors in terms of total citations.

Rank	Author	Records	Rank	Co-cited Author	Records
1	Abnous Khalil	17	1	Siegel RL	262
2	Taghdisi Seyed Mohammad	15	2	Mmeda H	182
3	Alibolandi Mona	14	3	Zhang Y	129
4	Ramezani Mohammad	13	4	Wang Y	113
5	Merlin Didier	10	5	Zhang L	105
6	Melguizo Consolacion	10	6	Liu Y	104
7	Kim Jong Oh	9	7	Chen Y	103
8	Villaverde Antonio	9	8	Li L	100
9	Vazquez Esther	9	9	Danhier F	94
10	Unzueta Ugutz	9	10	Li J	89

### 3.4 Journals and co-cited journals

**FIGURE 6:**
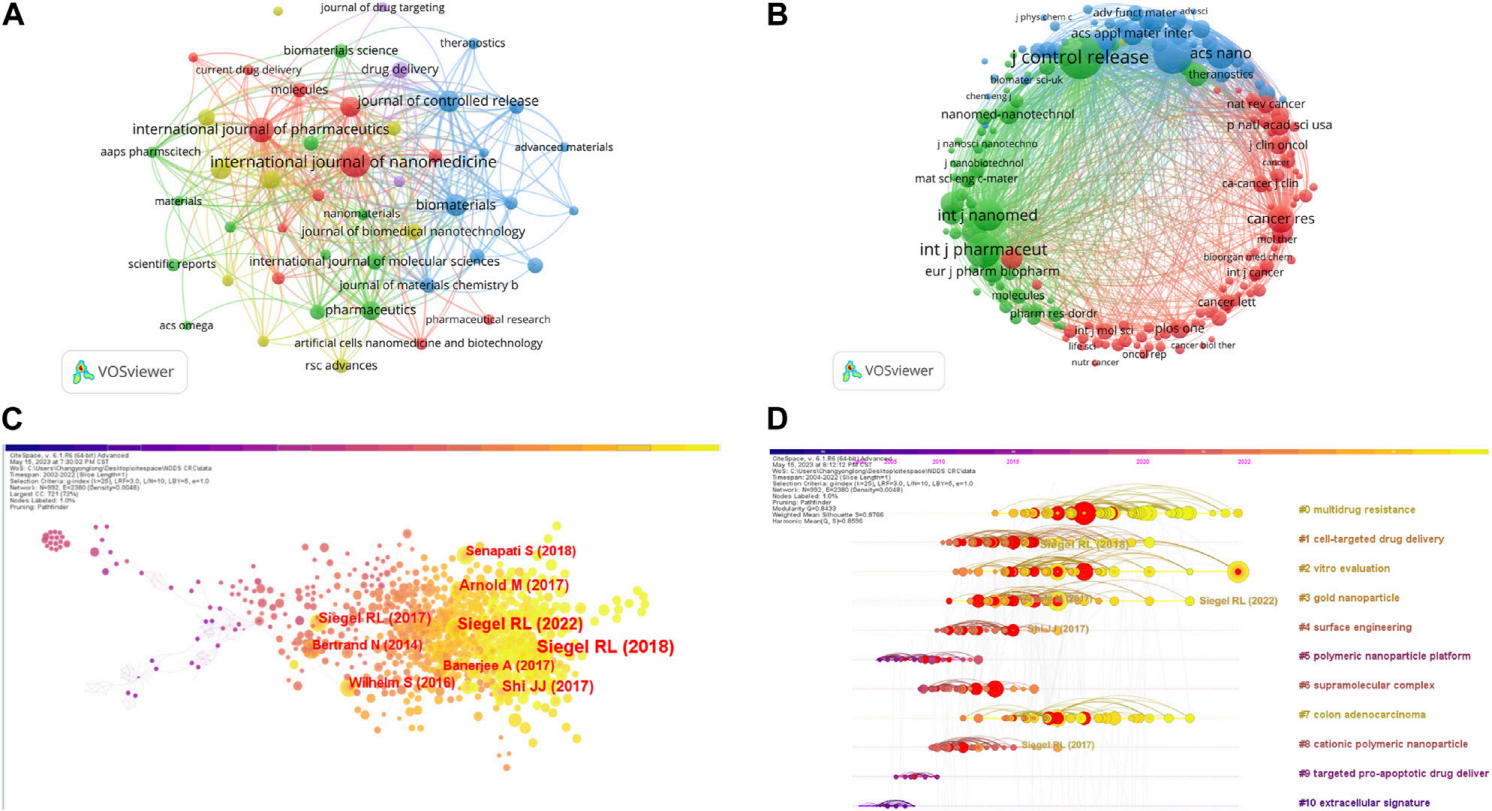
**(A)** Co-occurrence network diagram of journals generated by VOSviewer. **(B)** Co-cited network diagram of journals generated by VOSviewer. **(C)** The literature co-citation network, which was generated using CiteSpace. **(D)** Labels clustering of co-cited literature by CiteSpace based on LLR algorithm.

As presented in Table 4. Among the top 10 journals, The International Journal of Nanomedicine had the most publications (n = 85), followed by the International Journal of Pharmaceutics (n = 59) and Biomaterials (n = 45). Biomaterials also had the highest impact factor (IF = 15.304), and 90% of the journals had a JCR partition of Q1. We further filtered out 47 journals with a minimum of 10 relevant publications and formed 5 clusters. We created a journal co-occurrence network diagram as shown in Figure 6A, where larger nodes indicate a greater number of relevant publications. The connecting lines between nodes indicate a cross-citation relationship between two journals. It is notable that the journals in which NDDS in CRC research results are published have an active citation relationship.

**TABLE 4:** Top 10 journals and co-cited journals in terms of the number of published papers.

Rank	Journal	Records	Country	IF (2022)	Rank	Co-cite Journal	Records	Country	IF (2022)
1	International Journal of Nanomedicine	85	UK	Q1/7.033	1	Journal of Controlled Release	1294	Netherlands	Q1/11.467
2	International Journal of Pharmaceutics	59	Netherlands	Q1/6.510	2	Biomaterials	1156	UK	Q1/15.304
3	Biomaterials	45	UK	Q1/15.304	3	International Journal of Pharmaceutics	1000	Netherlands	Q1/6.510
4	Journal of Controlled Release	44	Netherlands	Q1/11.467	4	Advanced Drug Delivery Reviews	944	Netherlands	Q1/17.873
5	Journal of Drug Delivery Science and Technology	40	France	Q2/5.062	5	International Journal of Nanomedicine	897	UK	Q1/7.033
6	Colloids and Surfaces B: Biointerfaces	39	Netherlands	Q1/5.999	6	Cancer Research	780	United States	Q1/13.312
7	Pharmaceutics	36	Switzerland	Q1/6.525	7	ACS Nano	769	United States	Q1/18.027
8	International Journal of Biological Macromolecules	33	Netherlands	Q1/8.025	8	Colloids and Surfaces B: Biointerfaces	702	Netherlands	Q1/5.999
9	Carbohydrate Polymers	27	UK	Q1/10.723	9	Molecular Pharmaceutics	659	United States	Q1/5.364
10	Drug Delivery	27	United States	Q1/6.819	10	European Journal of Pharmaceutics and Biopharmaceutics	614	Netherlands	Q1/5.589

Table 4 also presents the top 10 co-cited journals, each cited over 500 times. The most cited journal was the Journal of Controlled Release (Co-citation = 1294), followed by Biomaterials (Co-citation = 1156) and the International Journal of Pharmaceutics (Co-citation = 1000). All top 10 co-cited journals were in JCR Q1 partition, with 5 having impact factors above 10. The highest impact factor was the ACS Nano (IF = 18.027). The top 10 most cited journals were published in America and Europe. Similarly, we mapped the journal co-citation network using journals with at least 100 co-citations. Figure 6B shows 192 journals included in three major clusters. Positive co-citation relationships were found among the Journal of Controlled Release, the International Journal of Pharmaceutics, and Cancer Research.

### 3.5 Analysis of co-cited references

In our study, a total of 79,644 references were cited in 1,866 publications. Table 5 presents the top 10 co-cited publications related to NDDS in the treatment of CRC. We utilized CiteSpace software to visualize the co-cited publications, with larger labels assigned to authors based on the number of citations, as depicted in Figure 6C. Notably, a publication by Professor Siegel RL from the United States on the American Cancer Society’s annual estimates and latest data on cancer incidence, mortality, and survival in the United States in 2018 received the highest number of citations, totaling 107. It is worth highlighting that the American Cancer Society provides annual estimates of new cancer cases and deaths in the United States and compiles the most recent data on population-based cancer incidence, which is valuable for epidemiological studies. Three out of the top 10 co-cited publications originate from this source. The journal CA: A Cancer Journal for Clinicians (IF 2022 = 286.130) had the highest impact factor among the top 10 co-citations, followed by Nature Reviews Cancer (IF 2022 = 69.800). The majority (70%) of the top 10 co-cited literature originated from the United States. We employed the LLR algorithm of CiteSpace’s automatic clustering function to label the co-citation network clusters, as depicted in Figure 6D. The analysis showed a modularity Q of 0.8433, indicating a strong clustering effect and a non-homogeneous network. The mean profile S reached a high level of 0.8596. The clusters are numbered in ascending order, starting from 0, and the numbers represent the number of studies contained within each cluster. The 79,644 references form 96 clusters, and in Figure 6D, we present the first 11 clusters as a time line, illustrating the evolving research hotspots over time. The largest cluster, named #0 multidrug resistance, indicates that numerous studies on CRC and NDDS have cited literature from this cluster. The clusters are numbered in ascending order, with smaller numbers indicating more studies within the corresponding cluster, thus highlighting the significance of multidrug resistance in this research area. The #5 cluster focuses on the polymeric nanoparticle platform as the initial topic of study in this field, while clusters #0 multidrug resistance, #2 *in vitro* evaluation, #3 gold nanoparticle, and #7 colon adenocarcinoma represent the current hotspots of research. There is an increasing trend of scientists focusing on nanomedicine in the context of colorectal cancer. Table 6 provides the top 25 most cited references. The rise in citations in this field began in 2010, and many of the co-cited references continue to be widely cited, indicating that NDDS remains a prominent research topic in CRC.

**TABLE 5:** Top 10 cited publications.

Rank	Co-cited reference	Total Citations	Centrality	Journal	IF (2022)	Corresponding author’s country
1	Cancer statistics, 2018	107	0.01	CA: a cancer journal for clinicians	Q1/286.130	USA
2	Cancer statistics, 2022	71	0.02	CA: a cancer journal for clinicians	Q1/286.130	USA
3	Cancer nanomedicine: progress, challenges and opportunities	51	0.07	Nature Reviews Cancer	Q1/69.800	USA
4	Global patterns and trends in colorectal cancer incidence and mortality	46	0.01	Gut	Q1/31.793	USA
5	Colorectal cancer statistics, 2017	41	0.04	CA: a cancer journal for clinicians	Q1/286.130	USA
6	Strategies for targeted drug delivery in treatment of colon cancer: current trends and future perspectives	33	0.02	Drug discovery today	Q1/8.369	India
7	Cancer nanotechnology: the impact of passive and active targeting in the era of modern cancer biology	33	0.04	Advanced drug delivery reviews	Q1/17.873	USA
8	Controlled drug delivery vehicles for cancer treatment and their performance	30	0.01	Signal transduction and targeted therapy	Q1/38.104	India
9	Colorectal cancer statistics, 2020	27	0.01	CA: a cancer journal for clinicians	Q1/286.130	USA
10	Nano based drug delivery systems: recent developments and future prospects	25	0.02	Journal of nanobiotechnology	Q1/9.429	Korea

**TABLE 6:** Top 25 References with the Strongest Citation Bursts.

Rank	References	Year	Strength	Begin	End	2002-2022
1	Anand P, 2010, BIOCHEM PHARMACOL, V79, P330, DOI 10.1016/j.bcp.2009.09.003	2010	6.16	2010	2015	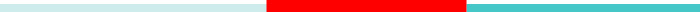
2	Shaikh J, 2009, EUR J PHARM SCI, V37, P223, DOI 10.1016/j.ejps.2009.02.019	2009	6.52	2012	2014	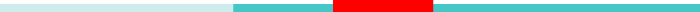
3	Wang AZ, 2012, ANNU REV MED, V63, P185, DOI 10.1146/annurev-med-040210-162544	2012	8.52	2014	2017	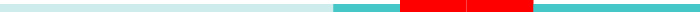
4	Allen TM, 2013, ADV DRUG DELIVER REV, V65, P36, DOI 10.1016/j.addr.2012.09.037	2013	6.47	2014	2018	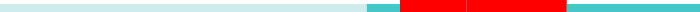
5	Bertrand N, 2014, ADV DRUG DELIVER REV, V66, P2, DOI 10.1016/j.addr.2013.11.009	2014	11.05	2015	2019	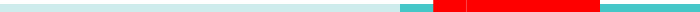
6	Anitha A, 2014, EUR J PHARM BIOPHARM, V88, P238, DOI 10.1016/j.ejpb.2014.04.017	2014	7.01	2015	2019	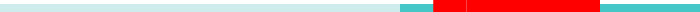
7	Barenholz Y, 2012, J CONTROL RELEASE, V160, P117, DOI 10.1016/j.jconrel.2012.03.020	2012	6.64	2015	2017	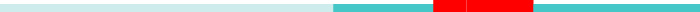
8	Mura S, 2013, NAT MATER, V12, P991, DOI 10.1038/NMAT3776	2013	7.83	2016	2018	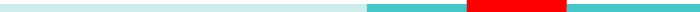
9	Anitha A, 2014, BBA-GEN SUBJECTS, V1840, P2730, DOI 10.1016/j.bbagen.2014.06.004	2014	6.47	2016	2019	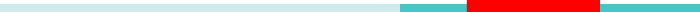
10	Sun TM, 2014, ANGEW CHEM INT EDIT, V53, P12320, DOI 10.1002/anie.201403036	2014	6.36	2016	2018	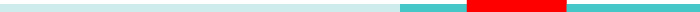
11	Torre LA, 2015, CA-CANCER J CLIN, V65, P87, DOI 10.3322/caac.21262	2015	5.92	2016	2020	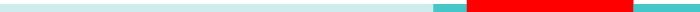
12	Xiao B, 2015, NANOSCALE, V7, P17745, DOI 10.1039/c5nr04831a	2015	7.69	2017	2019	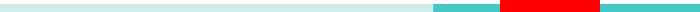
13	Maeda H, 2013, ADV DRUG DELIVER REV, V65, P71, DOI 10.1016/j.addr.2012.10.002	2013	6.73	2017	2018	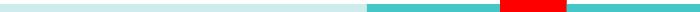
14	Siegel RL, 2022, CA-CANCER J CLIN, V72, P7, DOI 10.3322/caac.21708	2022	6.53	2022	2018	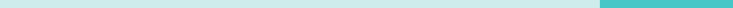
15	Wicki A, 2015, J CONTROL RELEASE, V200, P138, DOI 10.1016/j.jconrel.2014.12.030	2015	6.17	2017	2018	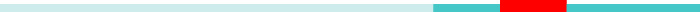
16	Hua S, 2015, NANOMED-NANOTECHNOL, V11, P1117, DOI 10.1016/j.nano.2015.02.018	2015	5.92	2017	2020	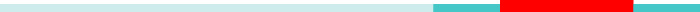
17	Xiao B, 2015, J MATER CHEM B, V3, P7724, DOI 10.1039/c5tb01245g	2015	5.92	2017	2020	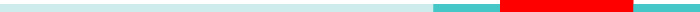
18	Wilhelm S, 2016, NAT REV MATER, V1, P0, DOI 10.1038/natrevmats.2016.14	2016	8.3	2018	2022	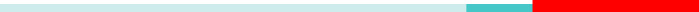
19	Blanco E, 2015, NAT BIOTECHNOL, V33, P941, DOI 10.1038/nbt.3330	2015	7.32	2018	2020	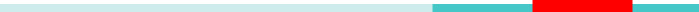
20	Siegel RL, 2017, CA-CANCER J CLIN, V67, P177, DOI 10.3322/caac.21395	2017	7.21	2018	2020	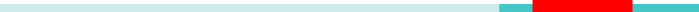
21	You XR, 2016, J MATER CHEM B, V4, P7779, DOI 10.1039/c6tb01925k	2016	5.93	2018	2020	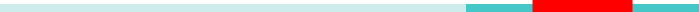
22	Shi JJ, 2017, NAT REV CANCER, V17, P20, DOI 10.1038/nrc.2016.108	2017	5.59	2018	2020	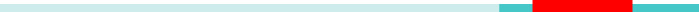
23	Arnold M, 2017, GUT, V66, P683, DOI 10.1136/gutjnl-2015-310912	2017	8.9	2019	2020	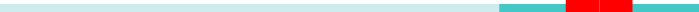
24	Cisterna BA, 2016, NANOMEDICINE-UK, V11, P2443, DOI 10.2217/nnm-2016-0194	2016	5.96	2019	2022	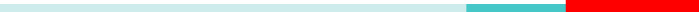
25	Siegel RL, 2018, CA-CANCER J CLIN, V68, P7, DOI 10.3322/caac.21442	2018	17.88	2020	2022	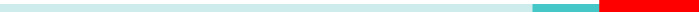

### 3.6 Co-cited keywords analysis

Keywords provide a concise summary of an article, and their analysis facilitates a deeper comprehension of the research topic. Moreover, high-frequency keywords often indicate the current research focal points in the field. This study examined 1,866 publications, resulting in 592 keywords after removing duplicates. Table 7 presents the 20 most frequent keywords. Additionally, we employed CiteSpace to generate the keyword co-occurrence network graph, keyword timeline graph, and identify the top 25 keywords with the highest citation intensity among the 1,866 publications. Table 7; Supplementary Figure S1A demonstrate that among the 592 keywords, the most frequently used keywords, excluding those from our search, were nanoparticle (n = 577), *in vitro* (n = 375), therapy (n = 215), cell (n = 207), and release (n = 207). This finding underscores their significance. The keyword timeline (Supplementary Figure S1B) clustering plot reveals the presence of five primary clusters: #0 oral delivery, #1 solid lipid nanoparticles, #2 cancer immunotherapy, #3 colorectal cancer, and #4 colon cancer. A further significant indicator of evolving research frontiers and research hotspots over time is the fluctuation in the intensity of keyword bursts. Supplementary Figure S1C illustrates that nanosphere, antitumor activity, and monoclonal antibody exhibit the highest strength values, implying their substantial citation frequency within a specific timeframe. These findings underscore the significance of these keywords and their correlation with research hotspots in the field. Furthermore, the citation bursts for keywords such as antibacterial (2019-2022) and breast (2020-2022) persist until 2022, with their intensity still undergoing changes, indicative of recent high attention received by certain research domains.

**TABLE 7:** Top 20 most frequent keywords analyzed using CiteSpace.

Rank	Keywords	Records	Centrality
1	drug delivery	724	0.04
2	nanoparticle	577	0.01
3	colorectal cancer	562	0.01
4	*in vitro*	375	0.02
5	colon cancer	368	0.06
6	delivery	318	0.02
7	therapy	215	0.02
8	cell	207	0.08
9	release	207	0.01
10	system	169	0.02
11	doxorubicin	160	0.04
12	chemotherapy	139	0.02
13	cancer	139	0.04
14	apoptosis	136	0.03
15	drug delivery system	132	0.05
16	*in vivo*	125	0.03
17	drug	120	0.01
18	breast cancer	111	0.05
19	gold nanoparticle	107	0.02
20	expression	107	0.02

### 3.7 Top 20 H-index authors and journals

The h-index was initially proposed by physicist Jorge E. Hirsch in 2005 (Hirsch, 2005). Hirsch introduced the h-index as a quantitative measure to assess the scientific output and impact of researchers (COSTAS and BORDONS, 2007). Since its inception, the h-index has gained widespread popularity. It is calculated by determining the highest number “h,” which signifies that a researcher has published at least “h” papers, each of which has received “h” or more citations. Currently, the h-index is extensively utilized in academia and research institutions to evaluate researchersʼ productivity and impact.

In this study, we employed the bibliometrix package of R to visualize the h-index of journals and authors. This analysis aimed to evaluate the authors and journals that published articles on NDDS in the CRC study. Table 8; Supplementary Figure S2A present the ranking of journals based on their h-index values. The International Journal of Nanomedicine secured the first position with an h-index of 34, closely followed by Biomaterials with an h-index of 27. Notably, the Journal of Controlled Release, despite not having a high number of publications among the top 10 journals, achieved the third rank in terms of h-index score. This suggests that the Journal of Controlled Release upholds rigorous quality standards for its articles.

**TABLE 8:** H-index scores of the top 20 journals.

Rank	Journal	h-index	Total citation
1	International Journal of Nanomedicine	34	3581
2	Biomaterials	27	2158
3	Journal of Controlled Release	27	1915
4	International Journal of Pharmaceutics	25	1891
5	Colloids and Surfaces B: Biointerfaces	22	1241
6	International Journal of Biological Macromolecules	20	892
7	ACS Nano	19	2113
8	Molecular Pharmaceutics	17	833
9	Carbohydrate Polymers	16	958
10	Journal of Drug Delivery Science and Technology	15	481
11	Pharmaceutics	15	516
12	Drug Delivery	14	510
13	Nanoscale	14	827
14	ACS Applied Materials & Interfaces	13	528
15	European Journal of Pharmaceutics and Biopharmaceutics	13	726
16	Nanomedicine	13	663
17	Journal of Biomedical Nanotechnology	12	579
18	Journal of Materials Chemistry B	12	541
19	Nanomedicine: Nanotechnology, Biology, and Medicine	12	629
20	Theranostics	12	635

Table 9; Supplementary Figure S2B display the top 20 authors according to their h-index scores. Li Y achieved the highest h-index of 14, followed by Melguizo C and Zhang Y, both with an h-index of 13.

**TABLE 9:** H-index scores of the top 20 authors.

Rank	Author	h-index	Total citation
1	Li Y	14	865
2	Melguizo C	13	468
3	Zhang Y	13	1040
4	Abnous K	12	542
5	Rameaani M	12	554
6	Wang Y	12	504
7	Alibolandi M	11	426
8	Cabeza L	11	288
9	Liu Y	11	412
10	Merlin D	11	730
11	Shieh MJ	11	444
12	Taghdisi SM	11	497
13	Jayakumar R	10	587
14	Ortiz R	10	314
15	Prados J	10	257
16	Unzueta U	10	353
17	Vazquez E	10	353
18	Villaverde A	10	353
19	Xiao B	10	653
20	Chen Y	9	261

### 3.8 Research trends evolution over the past 2 decades

Keywords assigned by authors to scientific publications are typically associated with the content of the publication and adequately reflect the main topical aspects of a particular field (Song et al., 2019). So, in order to conduct an extensive analysis of the prevailing themes using the keywords provided by the authors in the dataset, we utilized the Bibliometric package in R for visualization purposes. The analysis was carried out with specific parameters in place, encompassing a designated timeframe from 2002 to 2022, a minimum word frequency threshold of 5, a maximum of 5 words per year, and a word marker size of 5.

The thematic analysis involves examining clusters of authors' keywords and their interconnections to derive thematic patterns. These patterns are characterized by specific properties such as density and centrality. Density is depicted on the vertical axis, while centrality is represented on the horizontal axis. Centrality indicates the level of correlation among various topics, while density gauges the cohesion among the nodes (Song et al., 2019). These properties serve as measures to assess the development and significance of specific topics. Nodes with a higher number of connections within the thematic network demonstrate greater centrality, importance, and occupy crucial positions in the network. Similarly, the cohesiveness of a node, which signifies the density of a research field, indicates its capacity to evolve and sustain itself.

Noteworthy are the themes in the Q2 quadrant, including "inflammatory bowel disease," "oral administration," and "ulcerative colitis." Although these themes exhibit closer internal links, their contribution to the study of NDDS in CRC is relatively limited. These findings suggest that these topics need stronger connections with CRC and more linkage to enhance their potential impact. On the other hand, the theme of "active targeting" in the Q3 quadrant emerges as an emerging theme with limited relevance to the application of NDDS in CRC. However, Supplementary Figure S2C reveals that the themes "camptothecin," "targeted delivery," and "aptamer," situated between the Q1 and Q4 quadrants, have been thoroughly developed, indicating a more mature field. Theme analysis indicates the need for further development in areas such as "inflammatory bowel disease," "oral administration," "ulcerative colitis," and others. These topics have already reached a mature stage and hold significant potential for contributing to future research applications and the sustainable development of NDDS in CRC.

Similarly, we utilized the biblioshiny package to visualize the primary keywords in NDDS publications related to CRC research in the past 2 decades and conducted an analysis of research trends (Chang et al., 2023). Supplementary Figure S2D presents each topic as a line, with the circle denoting the most prevalent year for that particular topic. The size of the circle corresponds to the frequency of occurrence. Notably, we observed the emergence of new keywords such as "ulcerative colitis," "exosome," and "as1411"in the past 2 years, indicating their potential as current research hotspots. It is noteworthy that these findings align with the outcomes of our thematic analysis.

### 3.9 Country/region collaboration analysis

A total of 293 countries/regions made contributions to the collected publications. The collaboration network between countries/regions is depicted in Supplementary Figure S3B, with a minimum threshold of 3. In the figure, the red lines represent collaborative relationships between two countries. Thicker lines indicate closer collaborations. It is evident that the United States has established collaborative ties with numerous Asian and European countries in this research area. Furthermore, we employed biblioshiny to analyze the country/region information of the corresponding authors, as illustrated in Supplementary Figure S3B **.** China, with the highest number of publications, also holds the highest count of multinational publications (MCP), indicating its extensive partnerships with multiple countries/regions in this field.

## 4 Discussion

In this study, we conducted a comprehensive bibliometric analysis of colorectal cancer nanodrug delivery systems publications over the past 2 decades. Our analysis included quantitative, qualitative, and comprehensive assessments. After applying strict screening criteria, we identified 1,866 relevant publications from 293 countries/regions.

To gain insights into publication trends, we examined and analyzed various aspects, including authors and co-cited authors, institutions, journals, countries/regions, keywords, literature and co-cited literature, journal H-index, and author H-index. What’s more, we explored the evolution of research trends and potential future research areas in this field. Our study not only bridges a gap in the existing knowledge of NDDS in CRC but also provides valuable new insights into the advancement of this field in cancer research.

### 4.1 General information

Figure 3 demonstrates the progressive increase in publications on NDDS in CRC, showing a consistent year-on-year growth trend since the initial two publications in 2004. From 2004 to 2018, the growth in papers on NDDS in CRC research was relatively slow worldwide. However, in the last 5 years, there has been a rapid surge in publications. Therefore, this significant growth indicates that the field of NDDS in CRC is emerging and suggests a promising outlook for the future, implying that we may be entering a phase of remarkable advancements.

China has emerged as the leading global producer of publications in the field, surpassing all other countries/regions, thanks to the collective efforts of its universities and research institutions. Notably, 10 out of the top 20 active institutions are located in China. Interestingly, despite China’s overwhelming number of publications, the United States continues to dominate the field with the highest citation count and a significant presence of high H-index authors, which suggests the potentially greater impact of their articles. Recognizing this, the Chinese government and universities have implemented various measures to enhance the quality and impact of their academic publications. Professor Abnous Khalil from Mashhad University of Medical Sciences is the leading author in terms of the number of publications, with a research focus on aptamers, pharmacology, and nanotechnology. Professor Siegel RL from the American Cancer Society is the most cited author. Among the top journals, Biomaterials (IF 2022 = 15.304), Journal of Controlled Release (IF 2022 = 11.467), and Carbohydrate Polymers (IF 2022 = 10.723) are the three publications with an impact factor (IF) above 10, while the remaining seven journals have an IF between 5 and 10. Among the top 10 highly cited journals, five have an impact factor (IF) above 10, namely, Journal of Controlled Release (IF 2022 = 11.467), Biomaterials (IF 2022 = 15.304), Advanced Drug Delivery Reviews (IF 2022 = 17.873), Cancer Research (IF 2022 = 13.312), and ACS Nano (IF 2022 = 18.027).

### 4.2 Knowledge base

The term “citation bursts” signifies the timeframe when related research received a significant number of citations. This indicates the thorough scrutiny given by scientists to these documents, revealing the ongoing dynamics and emerging patterns in the field of NDDS in CRC research. Among the top 10 most cited publications, in 2010, Preetha Anand et al. (Anand et al., 2010). conducted a highly cited study where they employed polymer-based nanoparticles composed of poly(propylene-co-ethylene-glycol) (PLGA) and polyethylene glycol (PEG)-5000 for encapsulating curcumin. The resulting curcumin nanoparticles, named curcumin (NP), demonstrated efficient cellular uptake and exhibited significant effects in inducing apoptosis and inhibiting the proliferation of tumor cells. Animal studies also revealed that curcumin (NP) had superior bioavailability and a longer half-life compared to curcumin alone. This study underscores the potential of using PLGA nanoparticles to enhance the cellular uptake, bioactivity, and bioavailability of curcumin. It serves as a valuable reference for exploring enhanced bioavailability in other monomeric drugs as well. This was not the first study on curcumin, back in 2009, J. Shaikh et al. (Shaikh et al., 2009). from the Department of Pharmacy, National Institute of Pharmaceutical Education and Research (NIPER) in India, successfully improved the oral bioavailability of curcumin by formulating it into biodegradable nanoparticles. Through an emulsification technique, they achieved spherical nanoparticles with a size of 264 nm and an encapsulation rate of 76.9% at a 15% loading. These nanoparticles exhibited stability for 3 months under accelerated stability testing conditions for refrigerated products. X-ray diffraction analysis confirmed the amorphous nature of the encapsulated curcumin. *In vitro*, release studies demonstrated a diffusion-based release following the Higuchi pattern. Importantly, *in vivo*, pharmacokinetic analysis revealed a remarkable 9-fold increase in the oral bioavailability of curcumin compared to curcumin containing piperine. This finding strongly supports the potential of nanoparticles in enhancing the oral delivery of low bioavailability molecules like curcumin. What should be noted is that, generally, curcumin is often used as an adjuvant drug in the process of achieving an antitumor effect or in combination with other drugs to exert a synergistic effect. Therefore, the use of the nano preparation process is similar. Between the development of nanomedicine, Andrew Z (Wang et al., 2012). Wang and colleagues from the Lineberger Comprehensive Cancer Center at the University of North Carolina School of Medicine have authored a comprehensive review on the advancements in nanomedicine. Their review delves into different nanoparticle drug delivery platforms, highlights key concepts related to nanoparticle drug delivery, and presents a comprehensive overview of clinical data on approved nanoparticle therapies. Furthermore, the review discusses ongoing clinical studies in the field, shedding light on the current progress and future prospects of nanomedicine in healthcare. The field of cancer nanotherapeutics has witnessed exponential growth in research and development since the early 2000s. Nanoparticle technologies hold great promise in the commercialization of oncology drugs, offering improved efficacy and tolerability. Pharmaceutical companies have increasingly formed partnerships to harness proprietary nanoparticle technologies, hastening progress in the field. In 2014, Nicolas Bertrand published a comprehensive review in Advanced Drug Delivery Reviews that examined the lessons learned from the commercialization of first-generation nanomedicines like DOXIL^®^ and Abraxane^®^ (Allen and Cullis, 2013). The review delved into the current understanding of targeted and untargeted nanoparticles at various stages of development, including promising candidates like BIND-014 and MM-398. It addressed the opportunities and challenges encountered by nanomedicines in contemporary oncology, emphasizing the role of personalized medicine. The review covered essential topics such as the enhanced permeability and retention effects (EPR) and the mechanisms involved in preferential tumor "retention," such as active targeting, drug binding, and interactions with tumor-associated macrophages. The aim of the review was to enhance knowledge regarding the design and development of therapeutic nanoparticles for more effective applications in the field of cancer treatment. It is noteworthy that out of the top 10 most cited publications, 6 of them are reviews. This observation suggests that the field is still in its early stages, and researchers are placing greater emphasis on comprehensive reviews and summaries. The analysis of these co-cited articles can offer valuable insights that enhance our understanding of the progression of NDDS research in CRC treatment.

### 4.3 Emerging topics

In the realm of bibliometrics, the examination of frequently utilized keywords serves to highlight significant themes and emerging topic, offering valuable insights into the field’s development. Anticipating the trajectory of NDDS application in CRC research can be informed by identifying the most notable citation bursts associated with specific keywords. With this context, CiteSpace identified the following main areas of research in our study: “surface modification”, “tumor microenvironment”, “antibacterial peptides”, “produrg”. Surface modifications of NDDS can significantly affect cellular uptake and therapeutic efficacy (Naeem et al., 2020). For active targeting, the surface of the NDDS can be affixed with a targeting part such as an antibody or ligand, and the targeted part can bind specifically to an antigen or receptor overexpressed on the surface of the target cell, assisting in drug internalization to achieve avoidance of off-target effects (Banerjee et al., 2017). Currently, no specific description of surface modification in the field of CRC has been found. However, there are numerous reports of surface modification in the field of solid tumors. The application of surface modification in solid tumors can be categorized into two strategies (Li et al., 2020). The first strategy aims to disrupt the tumor microenvironment, thereby overcoming the defense mechanisms of solid tumors and enabling enhanced penetration of nanomedicines (Wang et al., 2018). This strategy can be further classified into two types: with or without exogenous energy supply (Chen et al., 2019). The second strategy involves comprehensive surface modification of nanodrugs to create suitable surface charge, softness, and other properties that can adapt to the tumor microenvironment and enable deeper tumor penetration (Wang et al., 2016). This strategy can also be classified into two types: non-bionic and bionic. Further details are presented in Supplementary Figure S3C. The tumor microenvironment (TME) plays a key role in tumorigenesis (Tang et al., 2021). Meanwhile, it is also becoming a strong and attractive therapeutic target in cancer therapy (Kim and Dang, 2006; Cairns et al., 2011). NDDS can fulfill the need to precisely target TME components and inhibit tumor progression through TME modulation. Compared to traditional therapeutic modalities, NDDS-transported drugs present a number of advantages, including prolonged circulation time, improved bioavailability, and reduced toxicity (Neri and Supuran, 2011). Recently, antimicrobial peptides (AMPs) have attracted significant attention as potential alternatives to conventional antibiotics, and bacteriocins within AMPs are expected to complement small-molecule antibiotics for cancer treatment (Neri and Supuran, 2011). However, bacteriocins have many limitations, such as bacteriocin resistance (Preciado et al., 2016). Addressing these challenges and improving the pharmacokinetics and pharmacodynamics of bacteriocins, NDDS is a promising avenue. Through the combination of bacteriocins with NDDS, it is possible to realize the full therapeutic potential of bacteriocins while bypassing their drawbacks (Sandgren et al., 2004). Such an approach holds significant promise for the treatment of solid tumors, including CRC. Prodrugs are compounds that remain inactive during transport, and the reactivation of their activity usually occurs in specific organs, tissues, or cells within the organism. For example, the covalent bond between the drug and the delivery vehicle can be cleaved in the tumor microenvironment, leading to the recovery of the prodrug. Specific chemical bonds can be introduced to develop prodrug delivery systems that stimulate responsiveness. Commonly used stimuli-responsive chemical bonds include hydrazone bonds, ester bonds, vinyl ether bonds, disulfide bonds, and peptide bonds sensitive to enzyme overexpression in tumor cells. Supplementary Figure S3D shows a schematic diagram of this system. Similar to other nano-delivery systems that are responsive to stimuli, stimuli-responsive prodrug delivery systems exhibit enhanced antitumor efficacy due to targeted delivery at the tumor site (Hu et al., 2019). It is worth mentioning that with the development of nanomedicine, the nanomaterials available to researchers are becoming more and more diverse, and the direction of research is becoming more and more precise (Fang et al., 2018; Ferreira-Faria et al., 2022). Recently, cellular nanocoating technology has emerged. This emerging biomimetic functionalization strategy takes advantage of the inherent ability of cells to interact with their environment, endowing traditional nanocarriers with enhanced biological properties, providing sustained circulation, and facilitating drug accumulation at the target site, enhancing the therapeutic effects of drugs while improving safety (Pereira-Silva et al., 2020). Currently, this technology has been applied to stem cells, cancer cells, platelets, and many other cell types in a variety of diseases including CRC (Zhang et al., 2022; Lopes et al., 2023; Zhu et al., 2023).

## 5 Innovations and limitations

In contrast to conventional methods, the employment of visual analysis tools such as CiteSpace, VOSviewer, and R offers a more comprehensive understanding of the dynamic research focus and trends pertaining to the association between NDDS and CRC. However, it is important to acknowledge the limitations of this study. The literature search was restricted to the core dataset of the WOS database, focusing solely on English publications, which might have led to the exclusion of relevant original literature. Consequently, the conclusions drawn may not be entirely comprehensive. To address these limitations, our future endeavors in this field aim to expand the scope of data collection and enhance our findings to provide valuable insights and support to researchers.

## 6 Conclusion

To the best of our knowledge, this study represents the first comprehensive analysis of publications on NDDS for CRC using bibliometric techniques (Teles et al., 2018). Our findings indicate that the current focus of NDDS research in CRC revolves around the utilization of nanomedicines with tailored surface modifications, targeting the tumor microenvironment, incorporating antibacterial peptides, and exploring the potential of produrgs. These areas represent the key hotspots and emerging trends in NDDS research for CRC. Nevertheless, we observed that despite the growing body of research, translation into clinical practice remains limited, with most NDDS technologies for CRC treatment being confined to animal experimentation. Moving forward, the field of nanomedicine, in conjunction with advancements in medicine, material science, and chemistry, holds promise for further propelling the application of NDDS in CRC treatment towards the realm of precision medicine. Overall, in this study, our findings are expected to provide a reference for expert decision-making and financial support.

## Data Availability

The original contributions presented in the study are included in the article/Supplementary Material, further inquiries can be directed to the corresponding author.

## Abbreviations

CRC, Colorectal cancer; NDDS, Nanodrug delivery systems; WOSCC, WOS Core Collection database; MCP, Multinational publications; PLGA, propylene-co-ethylene-glycol; NIPER, National Institute of Pharmaceutical Education and Research; TME, Tumor microenvironment; AMPs, antimicrobial peptides; IARC, International Agency for Research on Cancer.

## References

[B1] Allen T. M. Cullis P. R. (2013). Liposomal drug delivery systems: From concept to clinical applications. Adv. Drug Deliv. Rev. 65, 36–48. 10.1016/j.addr.2012.09.037 23036225

[B2] Anand P. Nair H. B. Sung B. Kunnumakkara A. B. Yadav V. R. Tekmal R. R. (2010). Retracted: Design of curcumin-loaded PLGA nanoparticles formulation with enhanced cellular uptake, and increased bioactivity *in vitro* and superior bioavailability *in vivo* . Biochem. Pharmacol. 79, 330–338. 10.1016/j.bcp.2009.09.003 19735646 PMC3181156

[B3] Arumov A. Trabolsi A. Schatz J. H. (2021). Potency meets precision in nano-optimized chemotherapeutics. Trends. Biotechnol. 39, 974–977. 10.1016/j.tibtech.2021.03.004 33832781 PMC10715812

[B4] Banerjee A. Pathak S. Subramanium V. D. Dharanivasan G. Murugesan R. Verma R. S. (2017). Strategies for targeted drug delivery in treatment of colon cancer: Current trends and future perspectives. Drug Discov. Today 22, 1224–1232. 10.1016/j.drudis.2017.05.006 28545838

[B5] Cairns R. A. Harris I. S. Mak T. W. (2011). Regulation of cancer cell metabolism. Nat. Rev. Cancer 11, 85–95. 10.1038/nrc2981 21258394

[B6] Chang Y. Ou Q. Zhou X. Liu J. Zhang S. (2023). Global research trends and focus on the link between colorectal cancer and gut flora: A bibliometric analysis from 2001 to 2021. Front. Microbiol. 14, 1182006. 10.3389/fmicb.2023.1182006 37213508 PMC10196369

[B7] Chen M. Wu J. Ning P. Wang J. Ma Z. Huang L. (2019). Remote control of mechanical forces via mitochondrial‐targeted magnetic nanospinners for efficient cancer treatment. Small 16, 1905424. 10.1002/smll.201905424 31867877

[B8] Choukaife H. Seyam S. Alallam B. Doolaanea A. A. Alfatama M. (2022). Current advances in chitosan nanoparticles based oral drug delivery for colorectal cancer treatment. IJN 17, 3933–3966. 10.2147/ijn.s375229 36105620 PMC9465052

[B9] Costas R. Bordons M. (2007). The h-index: Advantages, limitations and its relation with other bibliometric indicators at the micro level. J. Informetr. 1, 193–203. 10.1016/j.joi.2007.02.001

[B10] Fang R. H. Kroll A. V. Gao W. Zhang L. (2018). Cell membrane coating nanotechnology. Adv. Mater. 30, 1706759. 10.1002/adma.201706759 PMC5984176 29582476

[B11] Ferreira-Faria I. Yousefiasl S. Macário-Soares A. Pereira-Silva M. Peixoto D. Zafar H. (2022). Stem cell membrane-coated abiotic nanomaterials for biomedical applications. J. Control. Release 351, 174–197. 10.1016/j.jconrel.2022.09.012 36103910

[B12] Hirsch J. E. (2005). An index to quantify an individual’s scientific research output. Proc. Natl. Acad. Sci. U.S.A. 102, 16569–16572. 10.1073/pnas.0507655102 16275915 PMC1283832

[B13] Hu W.-W. Huang S.-C. Jin S.-L. C. (2019). A novel antimicrobial peptide-derived vehicle for oligodeoxynucleotide delivery to inhibit TNF-α expression. Int. J. Pharm. 558, 63–71. 10.1016/j.ijpharm.2018.12.082 30639220

[B14] Jung J. H. Chiang B. Grossniklaus H. E. Prausnitz M. R. (2018). Ocular drug delivery targeted by iontophoresis in the suprachoroidal space using a microneedle. J. Control. Release 277, 14–22. 10.1016/j.jconrel.2018.03.001 29505807 PMC5911252

[B15] Kaushik N. Borkar S. B. Nandanwar S. K. Panda P. K. Choi E. H. Kaushik N. K. (2022). Nanocarrier cancer therapeutics with functional stimuli-responsive mechanisms. J. Nanobiotechnol 20, 152. 10.1186/s12951-022-01364-2 PMC8944113 35331246

[B16] Kim J. Dang C. V. (2006). Cancer’s molecular sweet tooth and the warburg effect. Cancer Res. 66, 8927–8930. 10.1158/0008-5472.can-06-1501 16982728

[B17] Lammers T. Kiessling F. Hennink W. E. Storm G. (2012). Drug targeting to tumors: Principles, pitfalls and (pre-) clinical progress. J. Control. Release 161, 175–187. 10.1016/j.jconrel.2011.09.063 21945285

[B18] Li Z. Shan X. Chen Z. Gao N. Zeng W. Zeng X. (2020). Applications of surface modification technologies in nanomedicine for deep tumor penetration. Adv. Sci. 8, 2002589. 10.1002/advs.202002589 PMC7788636 33437580

[B19] Lopes D. Lopes J. Pereira-Silva M. Peixoto D. Rabiee N. Veiga F. (2023). Bioengineered exosomal-membrane-camouflaged abiotic nanocarriers: Neurodegenerative diseases, tissue engineering and regenerative medicine. Mil. Med. Res. 10, 19. 10.1186/s40779-023-00453-z 37101293 PMC10134679

[B20] Maulvi F. A. Desai D. T. Shetty K. H. Shah D. O. Willcox M. D. P. (2021). Advances and challenges in the nanoparticles-laden contact lenses for ocular drug delivery. Int. J. Pharm. 608, 121090. 10.1016/j.ijpharm.2021.121090 34530102

[B21] Naeem M. Awan U. A. Subhan F. Cao J. Hlaing S. P. Lee J. (2020). Advances in colon-targeted nano-drug delivery systems: Challenges and solutions. Arch. Pharm. Res. 43, 153–169. 10.1007/s12272-020-01219-0 31989477

[B22] Neri D. Supuran C. T. (2011). Interfering with pH regulation in tumours as a therapeutic strategy. Nat. Rev. Drug Discov. 10, 767–777. 10.1038/nrd3554 21921921

[B23] Oliveira C. de S. L. A. L. Schomann T. de Geus-Oei L.-F. Kapiteijn E. Cruz L. J. de Araújo Junior R. F. (2021). Nanocarriers as a tool for the treatment of colorectal cancer. Pharmaceutics 13, 1321. 10.3390/pharmaceutics13081321 34452282 PMC8399070

[B24] Pan X. Yan E. Cui M. Hua W. Han C. Zhi L. (2018). Skin perfusion pressure for the prediction of wound healing in critical limb ischemia: A meta-analysis. J. Informetr. 12, 481–487. 10.5114/aoms.2016.62220 PMC5949913 29765431

[B25] Pei Z. Chen S. Ding L. Liu J. Cui X. Li F. (2022). Current perspectives and trend of nanomedicine in cancer: A review and bibliometric analysis. J. Control. Release 352, 211–241. 10.1016/j.jconrel.2022.10.023 36270513

[B26] Peng C. Kuang L. Zhao J. Ross A. E. Wang Z. Ciolino J. B. (2022). Bibliometric and visualized analysis of ocular drug delivery from 2001 to 2020. J. Control. Release 345, 625–645. 10.1016/j.jconrel.2022.03.031 35321827

[B27] Pereira-Silva M. Santos A. C. Conde J. Hoskins C. Concheiro A. Alvarez-Lorenzo C. (2020). Biomimetic cancer cell membrane-coated nanosystems as next-generation cancer therapies. Expert. Opin. Drug. Deliv. 17, 1515–1518. 10.1080/17425247.2020.1813109 32812476

[B28] Poly T. N. Islam Md. M. Walther B. A. Lin M. C. Jack Li Y.-C. (2023). Artificial intelligence in diabetic retinopathy: Bibliometric analysis. Comput. Methods. Programs. Biomed. 231, 107358. 10.1016/j.cmpb.2023.107358 36731310

[B29] Preciado G. M. Michel M. M. Villarreal-Morales S. L. Flores-Gallegos A. C. Aguirre-Joya J. Morlett-Chávez J. (2016). Bacteriocins and its use for multidrug-resistant bacteria control. Elsevier, 329–349. 10.1016/b978-0-12-803642-6.00016-2

[B30] Radaic A. de Jesus M. B. Kapila Y. L. (2020). Bacterial anti-microbial peptides and nano-sized drug delivery systems: The state of the art toward improved bacteriocins. J. Control. Release 321, 100–118. 10.1016/j.jconrel.2020.02.001 32035192

[B31] Sandgren S. Wittrup A. Cheng F. Jönsson M. Eklund E. Busch S. (2004). The human antimicrobial peptide LL-37 transfers extracellular DNA plasmid to the nuclear compartment of mammalian cells via lipid rafts and proteoglycan-dependent endocytosis. J. Biol. Chem. 279, 17951–17956. 10.1074/jbc.m311440200 14963039

[B32] Shaikh J. Ankola D. D. Beniwal V. Singh D. Kumar M. N. V. R. (2009). Nanoparticle encapsulation improves oral bioavailability of curcumin by at least 9-fold when compared to curcumin administered with piperine as absorption enhancer. Eur. J. Pharm. Sci. 37, 223–230. 10.1016/j.ejps.2009.02.019 19491009

[B33] Siegel R. L. Miller K. D. Wagle N. S. Jemal A. (2023). Cancer statistics, 2023. CA A Cancer J. Clin. 73, 17–48. 10.3322/caac.21763 36633525

[B34] Song Y. Chen X. Hao T. Liu Z. Lan Z. (2019). Exploring two decades of research on classroom dialogue by using bibliometric analysis. Comput. Educ. 137, 12–31. 10.1016/j.compedu.2019.04.002

[B35] Tang L. Mei Y. Shen Y. He S. Xiao Q. Yin Y. (2021). Nanoparticle-mediated targeted drug delivery to remodel tumor microenvironment for cancer therapy. IJN 16, 5811–5829. 10.2147/ijn.s321416 34471353 PMC8403563

[B36] Teles R. H. G. Moralles H. F. Cominetti M. R. (2018). Global trends in nanomedicine research on triple negative breast cancer: A bibliometric analysis. IJN 13, 2321–2336. 10.2147/ijn.s164355 29713164 PMC5910795

[B37] van Eck N. J. Waltman L. (2009). Software survey: VOSviewer, a computer program for bibliometric mapping. Scientometrics 84, 523–538. 10.1007/s11192-009-0146-3 20585380 PMC2883932

[B38] Wang A. Z. Langer R. Farokhzad O. C. (2012). Nanoparticle delivery of cancer drugs. Annu. Rev. Med. 63, 185–198. 10.1146/annurev-med-040210-162544 21888516

[B39] Wang S. Huang P. Chen X. (2016). Hierarchical targeting strategy for enhanced tumor tissue accumulation/retention and cellular internalization. Adv. Mater. 28, 7340–7364. 10.1002/adma.201601498 27255214 PMC5014563

[B40] Wang X. Luo J. He L. Cheng X. Yan G. Wang J. (2018). Hybrid pH-sensitive nanogels surface-functionalized with collagenase for enhanced tumor penetration. J. Colloid Interface Sci. 525, 269–281. 10.1016/j.jcis.2018.04.084 29709781

[B41] Yang C. Merlin D. (2020). Lipid-based drug delivery nanoplatforms for colorectal cancer therapy. Nanomater. (Basel). 10, 1424. 10.3390/nano10071424 PMC7408503 32708193

[B42] Yang X. Xie Y. (2021). Recent advances in polymeric core–shell nanocarriers for targeted delivery of chemotherapeutic drugs. Int. J. Pharm. 608, 121094. 10.1016/j.ijpharm.2021.121094 34534631

[B43] Yu J. Feng Q. Wong S. H. Zhang D. Liang Q. Y. Qin Y. (2015). Metagenomic analysis of faecal microbiome as a tool towards targeted non-invasive biomarkers for colorectal cancer. Gut 66, 70–78. 10.1136/gutjnl-2015-309800 26408641

[B44] Zhang Z. Ji Y. Hu N. Yu Q. Zhang X. Li J. (2022). Ferroptosis-induced anticancer effect of resveratrol with a biomimetic nano-delivery system in colorectal cancer treatment. Asian J. Pharm. Sci. 17, 751–766. 10.1016/j.ajps.2022.07.006 36382309 PMC9640689

[B45] Zhao J.-F. Zou F.-L. Zhu J.-F. Huang C. Bu F.-Q. Zhu Z.-M. (2022). Nano-drug delivery system for pancreatic cancer: A visualization and bibliometric analysis. Front. Pharmacol. 13, 1025618. 10.3389/fphar.2022.1025618 36330100 PMC9622975

[B46] Zhu R. He H. Liu Y. Cao D. Yan J. Duan S. (2019). Cancer-selective bioreductive chemotherapy mediated by dual hypoxia-responsive nanomedicine upon photodynamic therapy-induced hypoxia aggravation. Biomacromolecules 20, 2649–2656. 10.1021/acs.biomac.9b00428 31125209

[B47] Zhu S. Li Z. Zheng D. Yu Y. Xiang J. Ma X. (2023). A cancer cell membrane coated, doxorubicin and microRNA co-encapsulated nanoplatform for colorectal cancer theranostics. Mol. Ther. Oncolytics 28, 182–196. 10.1016/j.omto.2022.12.002 36820302 PMC9937835

