# Supplementary material for: Mapping the intellectual structure and landscape of nano-drug delivery systems in colorectal cancer
Source: Front Pharmacol. 2023 Sep 14;14:1258937. doi: 10.3389/fphar.2023.1258937 (PMC10539472; doi:10.3389/fphar.2023.1258937)
Supplement: Supplementary file 1 [file DataSheet1.docx]

**
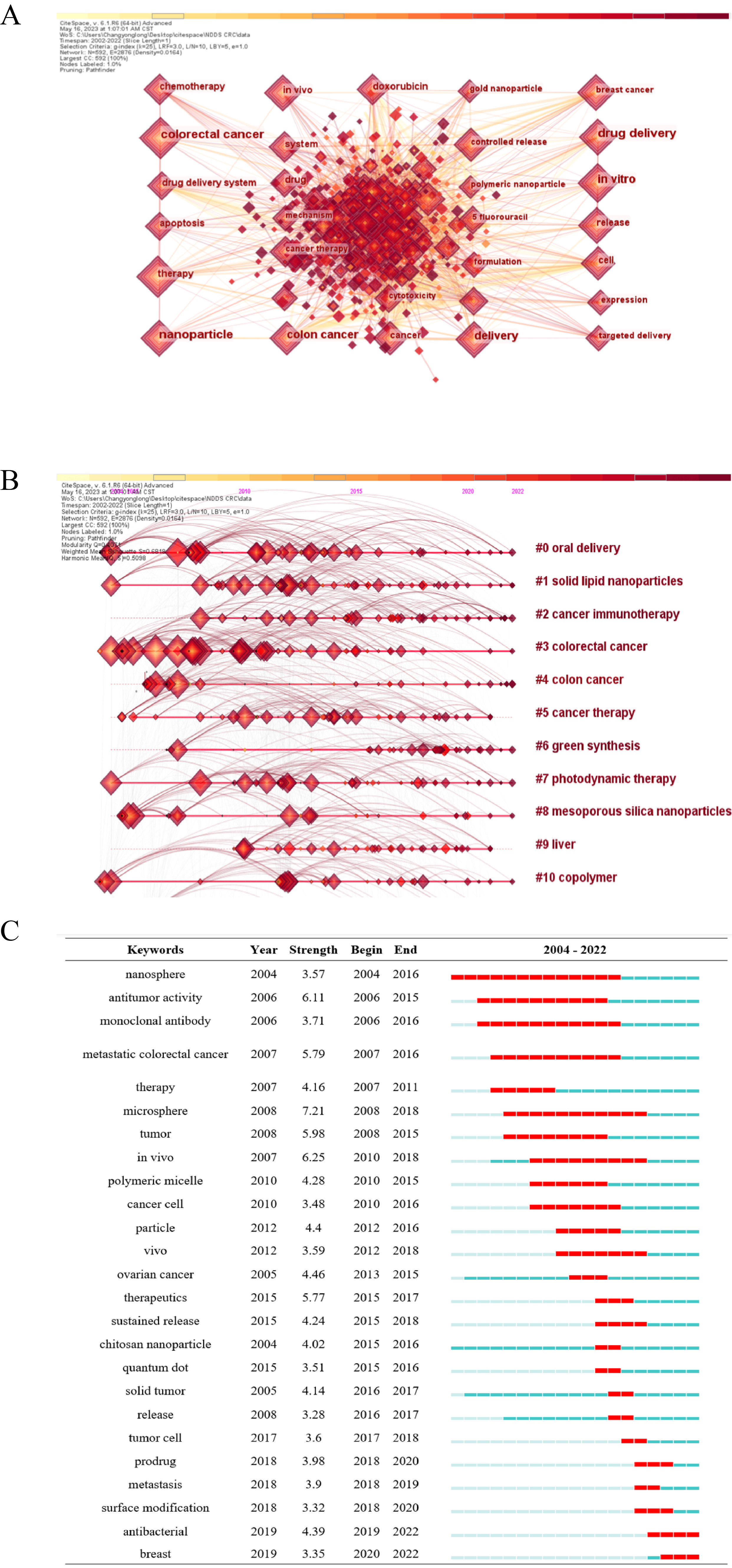
**

**Figure S1. Analysis of keyword co-occurrence. (A) Keyword co-occurrence network graph generated by CiteSpace (B) Keyword clustering timeline graph generated by CiteSpace. (C) Top 25 most cited keywords.**

**
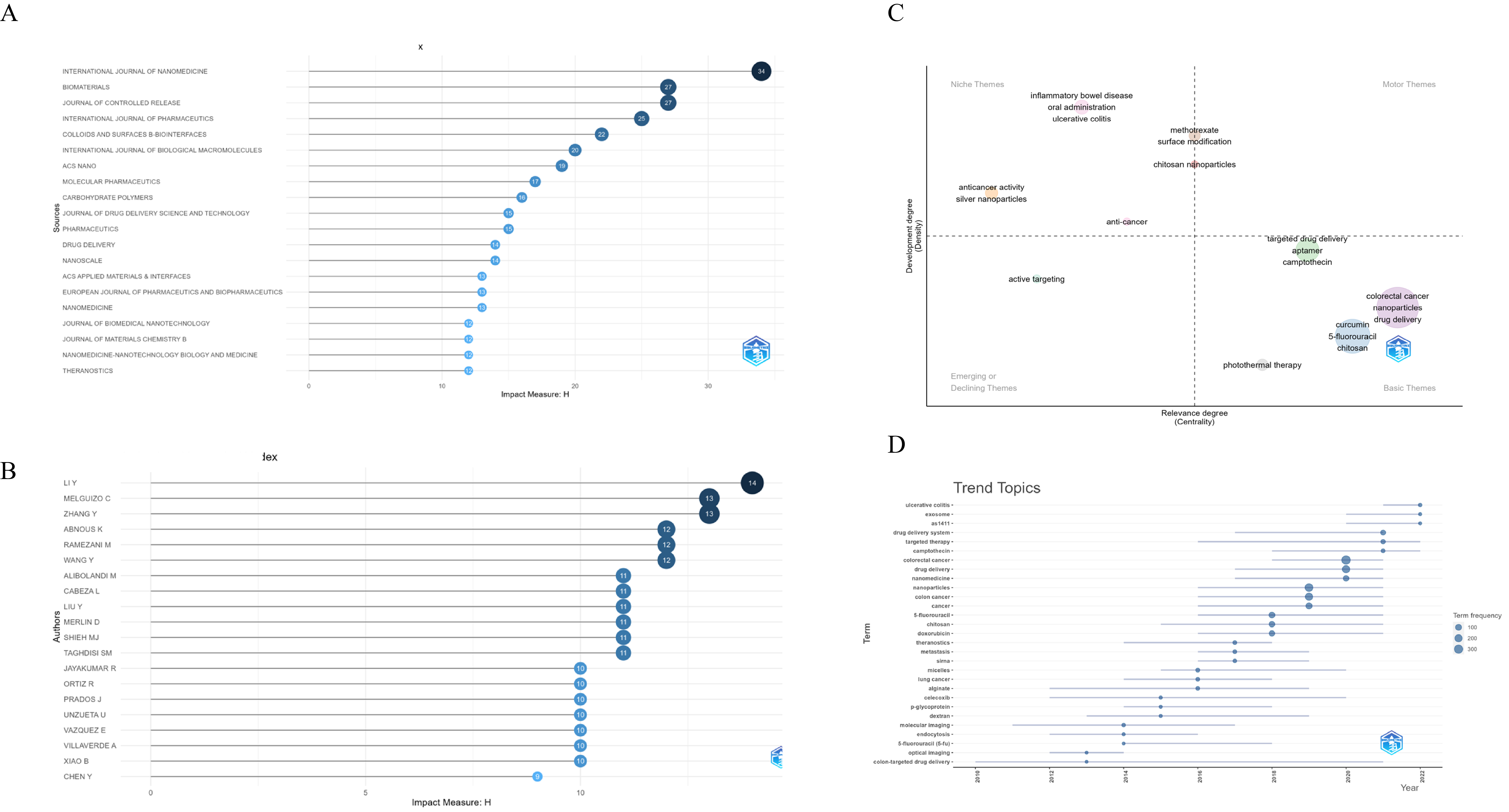
**

**Figure S2. (A) Ranking of published journals based on H-index. (B) Top 20 authors ranked by H-index. (C) Keyword-based research trend quadrant chart. (D) Keyword-based research theme trend map.**

**
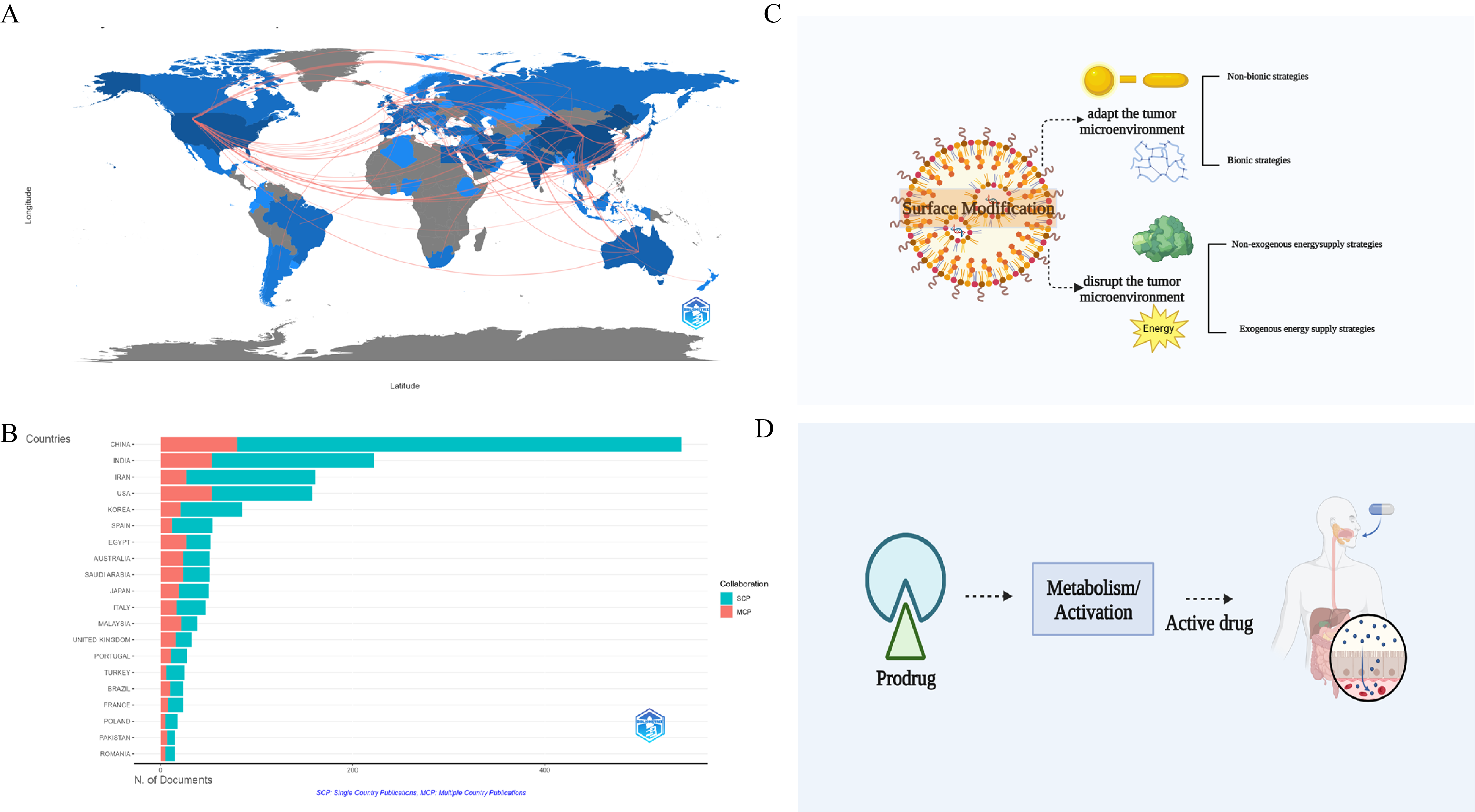
**

**Figure S3. (A) Global visualization map of publication collaborations, with red lines indicating collaborative relationships between countries/regions. (B) Country profiles of corresponding authors of publications generated by R-bibliometrix. (C) Schematic of NDDS surface modification classification. (D) Schematic diagram of prodrugs.**
